# Digital Standardized Patients: Conceptual Framework for Generative AI–Empowered Medical Education

**DOI:** 10.2196/91050

**Published:** 2026-08-20

**Authors:** Chuanlin Jia, Lulu Qi

**Affiliations:** 1 Publicity Department / Teacher Work Department, Zhongshan Hospital, Fudan University, No. 180, Fenglin Road, Xuhui District, Shanghai, Shanghai, China, 86 13764969722

**Keywords:** generative AI, virtual standardized patient, conversational AI, medical education, clinical communication simulation

## Abstract

Generative AI is driving medical education from digital support toward intelligent, interactive learning environments. The cultivation of doctor-patient communication skills requires not only technical proficiency but also communication competence, emotional sensitivity, and ethical judgment. This paper proposes a conceptual framework for AI-driven digital standardized patients (AI-SPs) to provide new approaches for communication training, humanistic education, and emotional engagement in medical curricula. This viewpoint integrates research findings from educational technology and medical education, elaborating the framework from 4 dimensions: system architecture, multimodal interaction, personality modeling, and ethical considerations. AI-SPs can provide adaptive, emotionally responsive interactions in repeatable, controllable simulated scenarios, enabling learners to experience diverse patient characteristics and clinical situations. The proposed “future learning” framework emphasizes personalization, contextualization, and reflective learning. During implementation, attention must be paid to data privacy, algorithmic bias, and human supervision. AI-SPs represent an extension of the traditional standardized patient model. They facilitate human-AI collaborative learning, support the cultivation of empathy, and provide a new pathway for the appropriate application of generative AI in medical education.

## Introduction

From multimedia instruction to online learning and then to virtual simulation, early educational technology innovations primarily expanded the coverage and efficiency of education, with technology itself positioned as an auxiliary tool. The emergence of generative AI has brought a qualitative change; it is not only a supporter of learning interactions but also an active participant [1].

AI is becoming a coconstructor of the learning experience. By responding to learners’ language, tone, and emotional cues, generative AI introduces the possibility of adaptive interaction. This is particularly crucial for medical education because the effectiveness of medical learning depends not only on cognitive accuracy but also on interpersonal sensitivity.

Medical education faces a long-standing challenge: how to ensure that students acquire high-quality clinical communication skills and humanistic care competencies while training physicians at scale? Communication training requires repeated practice, immediate feedback, and a safe environment for trial and error, areas in which traditional teaching methods have significant shortcomings. China’s medical education system faces this challenge with particular intensity. According to 2025 data from the National Center for Medical Education Development, China has 506 higher education institutions offering medical programs, including 112 independent medical colleges and schools, with a total of hundreds of thousands of medical students [2]. By geographic distribution, the eastern region has 240 institutions (47.4%), the central region 144 (28.5%), and the western region 122 (24.1%), presenting a pattern of “strong east, weak west” [2]. This uneven distribution of resources poses special challenges for the large-scale, balanced allocation of teaching resources such as standardized patients.

This paper proposes a conceptual framework for AI-driven digital standardized patients (AI-SPs) as an extension of existing simulation-based teaching to address the above challenges. The article first reviews the educational roles and limitations of traditional standardized patients (SPs), then elaborates on the core features and system architecture of AI-SPs, further proposes an integrated “future learning” framework, and finally discusses key ethical issues.

Regarding the technical pathway for developing AI-SP systems, current research has mainly followed 2 approaches. The first is a prompt engineering–based dynamic generation approach, in which carefully designed system prompts guide large language models (LLMs) to adopt specific patient roles and generate contextually appropriate responses. This approach provides advantages in role diversity and scenario adaptability, although maintaining role consistency remains challenging. The second is a fine-tuning–based standardization approach, which uses large-scale doctor-patient dialogue datasets to train models to reproduce specific communication patterns. While this approach improves output stability, it requires extensive, high-quality training data and may be less flexible in representing diverse patient personality types. Based on our experience in developing an AI-SP platform with multiple digital patient personas for medical student training, this framework adopts prompt engineering as the primary technical approach because it aligns with the educational objective of providing diverse and customizable patient roles for communication training.

## Background and Concept Analysis

### Value and Limitations of Traditional SPs

SPs were first introduced by Barrows [3] in 1963, marking an important shift in medical education toward experiential, learner-centered approaches. SPs enable learners to practice diagnostic reasoning, communication strategies, and professional behaviors in a safe, controlled environment [4].

SPs have become a core tool for clinical skills training and assessment, including objective structured clinical examinations (OSCEs). Their value lies in providing a controlled training environment, supporting reflective feedback, and cultivating a patient-centered perspective. However, SP-based teaching faces persistent challenges: high operational costs, limited scalability, significant variability in performance, and pronounced emotional fatigue, particularly in large-scale or resource-constrained educational settings.

These limitations are especially evident in communication-intensive scenarios such as preoperative conversations, breaking bad news, and managing patients who are emotionally distressed, precisely the scenarios that require the highest degree of consistency and repeatability in teaching.

### Comparison Between AI-SP and Related Concepts

To clearly define the concept proposed in this paper, it is necessary to distinguish it from existing related concepts. Table 1 compares traditional SPs, rule-based virtual patients, general-purpose chatbots, and the AI-SP proposed in this paper.

**Table 1.** Comparison of different standardized patient (SP)–related concepts^a^.

Feature dimension	Traditional SP (human performer)	Rule-based virtual patient	General chatbot	Proposed AI-SP^b^
Interaction modality	Face-to-face with real person	Menu selection and scripted dialogue	Free-text dialogue	Text or voice switching+virtual avatar
Response generation	Script-based	Preprogrammed rules	General generation	Context-sensitive generation+affective computing
Personality consistency	Depends on actor performance	None or fixed	Unstable	Based on Big Five personality model and Satir communication stance theory
Emotional dynamics	Simulatable but difficult to standardize	None	None or superficial	Emotional state can be influenced by student behavior and evolve in real time
Scalability	Low (high training cost and long cycle)	High	High	High (parameterized customization)
Repeatability	Moderate (affected by actor condition)	High	Low	High (controllable and recordable)
Real-time feedback	Depends on post hoc comments from teachers or actors	None or limited	None	Can generate formative assessment reports based on communication frameworks
Applicable scenarios	OSCE^c^ and small-class teaching	History-taking training	General Q&A^d^	Communication training, humanistic education, and emotionally intense scenarios

a Rule-based virtual patients refer to early digital patient simulators based on predefined scripts.

b AI-SP: AI-driven digital standardized patient.

c OSCE: objective structured clinical examination.

d Q&A: question and answer.

The comparison in Table 1 shows that the AI-SP is a pedagogical agent with stable personality, affective responsiveness, multimodal interaction support, and parameterized customization, rather than a simple “chatbot playing the role of a patient” [5].

### Specificity of the Chinese Medical Education Context

This paper focuses on the Chinese medical education context based on 3 main considerations.

#### Imbalance in Scale and Resources

China’s 506 medical education institutions are distributed across regions with significantly different resources. Establishing and maintaining professional SP programs requires sustained financial investment and systematic training, which poses substantial barriers for institutions in central and western China. As a scalable digital solution, the AI-SP has the potential to partially alleviate this resource imbalance, provided that institutions have basic information technology infrastructure for deployment.

#### Specificity of Cultural and Communication Patterns

Cross-cultural research has revealed significant differences in doctor-patient communication patterns across cultural contexts. A comparative study by Liu et al [6] involving 500 patients from China and 500 from the United Kingdom found that Chinese patients reported significantly lower scores on communication quality, decision-making participation, and emotional expression compared to British patients. The study further identified power distance and collectivist orientation as key predictors of these differences: higher power distance was associated with lower communication quality, reduced patient participation, and more restrained emotional expression. This means that when designing AI-SPs for the Chinese context, Western models cannot simply be replicated; rather, they must be adapted to local communication norms and patient expectations.

In addition, Chinese medical humanities education has recently begun to systematically reflect on indigenous traditions. The 2025 “Life Has Pain, Apricot Has You” Chinese medical humanities expert consensus [7] explicitly states that Chinese medical humanities education has long drawn primarily from Western concepts, with insufficient transmission of the traditional Chinese medical humanistic spirit. Rooted in the historical allusion to Dong Feng’s “apricot forest” from the Eastern Han Dynasty, this consensus articulates a medical humanistic spirit grounded in traditional Chinese culture that also meets contemporary needs, emphasizing physician-patient empathy as a bond. This consensus provides a cultural reference for AI-SP design: it should not only merely simulate “what the patient says” but also embody the traditional expectation of physician-patient mutual trust in the Chinese context.

#### Support From the Policy Environment

China has issued multiple national policies encouraging the integrated development of “AI+education” and “AI+healthcare”: the New Generation Artificial Intelligence Development Plan (State Council Document [2017] number 35) lists intelligent education as a key area; the 14th Five-Year Plan for Digital Economy Development promotes the construction of new educational infrastructure; in 2025, the National Health Commission and 4 other departments jointly issued the Implementation Opinions on Promoting and Regulating the Application of “AI+Healthcare,” identifying 24 key applications including research and teaching; and in the same year, the State Council issued the Opinions on Deeply Implementing the “AI+” Action, promoting the exploration of AI applications in health care scenarios. These policies provide macroenvironmental support for the research, development, and application of AI-SPs.

In summary, the Chinese context is important because it simultaneously presents large-scale demand, unique cultural communication patterns, and a clear national policy direction, 3 factors that together constitute the practical foundation for AI-SP development.

## Conceptual Framework of AI-SP

### Definition and Core Features

This paper defines AI-SP as a digital interactive agent driven by generative AI, possessing stable personality traits and affective responsiveness, capable of engaging in multimodal, context-sensitive natural conversations with medical learners in repeatable, controllable simulated environments, and supporting parameterized configuration of clinical scenarios. Unlike general-purpose chatbots or rule-based virtual patients, it has the following five core features:

1. Multimodal interaction: it supports real-time switching between text and voice dialogue, with a virtual avatar. This design brings interactions closer to real-world doctor-patient communication scenarios, allowing learners to choose interaction methods that suit their learning preferences.
2. Personality consistency: based on the Big Five personality model and Satir communication stance theory, AI-SP maintains stable personality traits and emotional response patterns across multiple independent interactions (eg, persistent anxiety triggered by high neuroticism and defensive blame caused by low agreeableness). This mechanism effectively avoids the role drift and logical conflicts common in generative AI, ensuring that the virtual patient maintains a unified cognitive framework and emotional tone during extended dialogues [8], thereby constructing a highly realistic teaching environment with stable feedback loops.
3. Affective dynamic responsiveness: AI-SP’s emotional state is not a fixed script but can be influenced by learners’ communication behaviors (eg, tone, wording, and empathic expression) and evolve in real time. For example, when a learner asks questions in a blunt or empathy-deficient manner, AI-SP may exhibit anxiety or distrust; conversely, appropriate empathic expression may alleviate its negative emotions.
4. Parameterized customization: teachers can quickly generate diverse patient cases by adjusting a series of parameters, including patient demographic characteristics (age, gender, occupation, and education level), emotional states (ranging from calm to anxious, angry, and sad, etc), personality traits (scores on the Big Five dimensions), clinical scenarios (disease type, severity, and whether they involve bad-news disclosure), and social factors (family support, financial burden, and so on).
5. Real-time feedback and reflective support: after the interaction, the system can generate formative assessment reports based on communication skills frameworks (eg, the SEGUE [Set the Stage, Elicit Information, Give Information, Understand the Patient’s Perspective, and End the Encounter] Scale and the SPIKES [setting up the interview, assessing the patient’s perception, obtaining the patient’s invitation, giving knowledge and information, addressing empathy and emotions, strategy and summary] bad-news disclosure model), identifying the learner’s strengths and weaknesses in communication and suggesting directions for improvement.

### Technical Implementation Mechanisms Underlying the Educational Functions of AI-SP

This section further describes the specific technical mechanisms underlying the educational functions of AI-SPs to clarify the operational feasibility of the proposed framework.

The current AI-SP system primarily adopts a prompt engineering–based approach, supplemented by retrieval-augmented generation (RAG) and agent-based architectures, although model fine-tuning has not yet been implemented. Table 2 summarizes the correspondence between key educational functions of AI-SPs and their underlying technical implementations.

**Table 2.** Mapping of AI-driven digital standardized patients (AI-SPs) educational functions to implementation approaches and underlying technical mechanisms^a^.

Educational function	Implementation in the platform	Underlying technical mechanism
Personalized patient personas (eg, normal, anxious, challenging, and angry)	Before training, users select a patient type, and the system invokes the corresponding persona-specific prompt template.	Prompt engineering+intrinsic LLM^b^ capabilities: persona profiles are constructed for different patient types, with emotional characteristics, communication preferences, and behavioral rules defined within system prompts. The contextual understanding capability of LLMs is used to generate continuous dialogues that are consistent with predefined personality characteristics.
Language style and expression patterns (eg, colloquial language and differences in educational background)	Controlled through prompt constraints.	Prompt engineering: personality characteristics, educational background, and other contextual factors are incorporated as prompt parameters to constrain language style and the use of medical terminology.
Dynamic emotional responsiveness (eg, patients becoming less distressed after receiving empathic responses from learners)	Supported through LLM-based contextual understanding combined with prompt constraints.	Prompt engineering+intrinsic LLM capabilities: emotional transition rules for different patient personas are predefined within system prompts. Through multiturn dialogue processing and contextual reasoning, LLMs maintain consistency in emotional evolution and response logic throughout interactions.
Generation and customization of clinical scenarios (eg, adjustment of patient age, disease condition, and social background by educators)	Prompt templates contain configurable variables. Users input clinical departments, diagnoses, and intended communication scenarios, which are dynamically assembled by the backend into complete persona prompts.	Prompt engineering: structured user-defined parameters are dynamically injected into system prompts. LLMs infer patient illness perceptions, major concerns, and communication goals based on these parameters. Demographic and cultural factors (eg, age and educational background) can be incorporated as expandable prompt variables.
Real-time feedback and scoring (eg, SEGUE^c^-based communication assessment after interaction)	A separate AI agent is used to analyze the completed dialogue.	RAG^d^+AI agent architecture: SEGUE assessment criteria are structured into a knowledge base. The agent retrieves relevant assessment criteria through RAG during evaluation and applies LLM-based semantic analysis to the complete dialogue, enabling item-level evaluation, evidence identification, and score generation.

a The current system primarily adopts prompt engineering rather than model fine-tuning for several reasons. First, the system is designed for dynamic role-playing rather than standardized dialogue generation, making prompt engineering a more suitable approach at the current stage. Second, fine-tuning requires large-scale standardized doctor-patient dialogue datasets and substantial development resources, which remain challenging during early-stage implementation. With the accumulation of sufficient interaction data in the future, fine-tuning may serve as a potential approach to further enhance the realism and fidelity of patient personas.

b LLM: large language model.

c SEGUE: Set the Stage, Elicit Information, Give Information, Understand the Patient’s Perspective, and End the Encounter.

d RAG: retrieval-augmented generation.

### System Architecture

This paper aims to propose a conceptual framework rather than complete technical specifications, so only the key modules and their functions are described. The system architecture comprises four core modules:

1. LLM core: as the “brain” of the system, it generates natural language responses consistent with clinical scenarios and patient roles. In the current AI-SP system, this module is optimized primarily through medical dialogue–specific prompt engineering to improve medical accuracy and role consistency of generated responses. The output of this module is controlled through prompt engineering, which provides the technical foundation for parameterized clinical scenario generation and for constraints on patient-specific language styles. This module serves as the core computational component enabling context-sensitive dialogue generation and the integration of other AI-SP functions.
2. Affective computing module: responsible for identifying emotional cues in learner input (eg, tone, keywords, and empathic expression) and calculating the affective response the AI-SP should present based on the current patient’s personality traits and emotional state. Emotional states are modeled as continuous processes rather than fixed scripted reactions, allowing learners’ communication behaviors to influence the trajectory of the patient’s emotional evolution. This module serves as the core mechanism enabling the dynamic emotional responsiveness function of the AI-SP system.
3. Personality and communication stance modulation module: this module integrates the Big Five personality model and Satir communication stance theory. The former defines the patient’s stable personality traits (eg, agreeableness and neuroticism), while the latter defines typical communication behavior patterns under pressure (eg, placating, blaming, superreasonable, irrelevant, and congruent). Together, they determine AI-SP’s response tendencies to different communication strategies. For example, highly neurotic patients are more likely to amplify negative information and tend toward a “blaming” stance, while highly agreeable patients are more likely to adopt a “congruent” or “placating” stance, cooperating with the learner’s inquiry. This module serves as the core mechanism enabling personalized patient persona modeling within the AI-SP system.
4. Safety guardrail and content filtering module: this model ensures that the system’s responses do not contain inappropriate content, do not provide medical advice beyond the SP role, and do not respond to learners in educationally unsuitable ways. For sensitive scenarios involving patient safety or ethical boundaries, the system should incorporate a “human-in-the-loop” mechanism, where the teacher can intervene and correct when necessary. This module provides essential safety assurance for all educational functions of the AI-SP system.

Based on the above architecture, our research team has preliminarily developed an AI-SP prototype system that supports both text and voice interaction modes and integrates a virtual avatar module. The detailed technical architecture and workflow of this prototype system are shown in the Multimedia Appendix 1.

### Theoretical Foundations of Teaching

The design of AI-SP is based not only on technical feasibility but also on clear pedagogical theoretical support [9]. Two core theories are briefly explained below:

1. Kolb’s experiential learning cycle: Kolb’s experiential learning theory holds that effective learning comprises 4 stages: concrete experience → reflective observation → abstract conceptualization → active experimentation. This theory has been widely applied in medical simulation teaching and research. AI-SP can fully embody this cycle: learners gain near-realistic communication experiences through simulated consultations with the system; the system generated feedback reports help learners review their communication behaviors and their effects; learners internalize the feedback into understanding communication principles; and then attempt improved communication strategies in the next simulated scenario. This cycle can be repeated at low cost and high frequency, which is difficult to achieve with traditional SPs.
2. Deliberate practice: Psychologist Ericsson’s (1993) deliberate practice theory emphasizes that skill improvement requires clear goals, immediate feedback, sufficient repetition, and gradually increasing difficulty. AI-SPs can provide technical support for this theory in several ways: learners can select specific communication skill goals (eg, “practice empathic expression” or “practice bad news disclosure”); the system provides immediate, consistent feedback; the same scenario can be repeated unlimited times; teachers adjust scenario parameters based on learner progress, gradually increasing complexity. Existing empirical studies have shown that AI-SPs designed with structured information architectures can achieve noninferiority to human SPs in communication skill improvement, with unique advantages for self-efficacy [10].

### Technical Challenges and Coping Strategies

Acknowledging existing technical limitations, this section focuses on three key technical challenges and proposes corresponding improvement strategies and solutions:

1. AI “hallucination”: generative AI may produce factually inaccurate information when uncertain. In medical education scenarios, this may manifest as the system describing nonexistent symptoms or providing statements that are inconsistent with clinical guidelines. The coping strategy is to use RAG technology to constrain the system’s responses within preapproved clinical knowledge bases. For high-stakes assessment scenarios, a “human-in-the-loop” supervision mechanism should be maintained.
2. Inconsistency in affective responses: although the system is designed with personality modulation and affective computing modules, inconsistent affective responses may still occur during long dialogues or multiturn interactions. The coping strategy is to introduce a dialogue state tracking mechanism into the system design to ensure consistency in personality and emotional state throughout the conversation. Additionally, teachers should conduct spot-check validation during the initial deployment phase.
3. Cultural sensitivity and content safety: the system’s responses may inadvertently reflect stereotypes or culturally insensitive expressions toward specific populations (eg, patients from different regions, ages, or educational backgrounds). The coping strategy is to incorporate doctor-patient communication norms and humanistic consensus from the Chinese context into training data and prompt design and to establish a regular review mechanism involving medical humanities experts to assess the appropriateness of AI-SP responses.

It should be noted that the AI-SP proposed in this paper is not intended to replace real patient contact. Its value lies in providing learners with preparation, reflection, and supplementation—safe, controllable, repeatable practice opportunities before contact with real patients, and reflective support after real patient contact.

## “Future Learning” Framework: Educational Integration of AI-SP

This section further proposes an integrated “future learning” framework to explain how this system can be integrated into medical education practice.

### Three Core Dimensions of the Framework

This framework is based on three core dimensions:

1. Personalization: the system can adjust the complexity and support level of interactions according to the learners’ ability levels. It provides more structured prompts and guided follow-up questions for junior medical students while presenting more ambiguous and complex patient statements for senior students, thereby forcing them to apply clinical reasoning skills.
2. Contextualization: teachers can quickly generate patient cases covering a wide range of clinical scenarios through parameterized configurations. Configurable variables include patient demographic characteristics, disease type and severity, emotional state, personality traits, and sociocultural background. This capability enables the system to simulate scenarios that are difficult to standardize in traditional SP training (eg, cross-cultural communication, patients with specific religious beliefs).
3. Emotional engagement: the system’s affective dynamic responsiveness forces learners to face “emotional” patients. Learners’ communication behaviors (especially empathic expression) directly affect patients’ emotional states and cooperation. This design incorporates “emotional labor” into the core of communication training—learners must not only say the right things but also express emotions appropriately at the right moments.

### Integration With Existing Teaching Systems

AI-SP is not intended to replace existing SP programs but to serve as their supplement and extension. Three possible integration pathways are described below:

1. Standardized practice before the OSCE: before formal assessments, learners can repeatedly practice the same clinical scenario to become familiar with communication processes and common coping strategies. This helps reduce assessment anxiety and ensures that all learners receive basic exposure training.
2. Supplement and expansion of SP resources: for resource-limited medical schools, AI-SP can serve as an “always available” alternative, covering high-frequency practice that is difficult to achieve with human SPs due to cost or complexity. For schools with mature SP programs, the system can be used to simulate special or rare clinical scenarios.
3. Data foundation for formative assessment: all interactions can be completely recorded and analyzed. These data can provide learners with detailed personal communication skill profiles, provide teachers with class-level analysis of common problems, and provide data-based decision support for curriculum improvement.

### Evaluation Research Framework

This paper aims to propose a conceptual framework rather than present empirical data. Therefore, we propose the following research framework based on Kirkpatrick’s 4-level evaluation model to guide future empirical validation (Table 3).

**Table 3.** AI-driven digital standardized patients (AI-SPs) evaluation framework based on the Kirkpatrick model^a^.

Evaluation level	Evaluation content	Measurement tools	Time frame
Level 1: reaction	Learner satisfaction, perceived usefulness, and willingness to use	Self-designed questionnaire and system usage logs	Short term (1‐3 mo)
Level 2: learning	Knowledge acquisition and skill improvement	SEGUE^b^ Scale, JSPE^c^ empathy scale, and pre-post test design	Medium term (3‐6 mo)
Level 3: behavior	Transfer of communication behaviors to clinical practice	Comparison of AI-SP^d^ training group vs control group in OSCE^e^ and clinical supervisor evaluation	Medium to long term (6‐12 mo)
Level 4: results	Ultimate impact on patient outcomes	Patient satisfaction in real clinical settings and communication-related medical error rates	Long term (>12 mo)

a Kirkpatrick’s 4-level evaluation model was proposed by Donald Kirkpatrick in the 1950s and is a widely used training effectiveness evaluation framework in medical education research. The SEGUE Scale is used to assess doctor-patient communication skills; the JSPE Scale is used to measure empathy; and OSCE stands for the Objective Structured Clinical Examination.

b SEGUE: Set the stage, Elicit information, Give information, Understand the patient’s perspective, and End the encounter—a framework for teaching and assessing communication skills.

c JSPE: Jefferson Scale of Physician Empathy.

d AI-SP: AI-driven digital standardized patient.

e OSCE: objective structured clinical examination.

Preliminary empirical studies provide feasibility support for this roadmap. Yang et al [11] identified high-learning–outcome pathway combinations in AI-generated content–enabled situational teaching using the fuzzy-set qualitative comparative analysis methodology; Gao et al [12] identified 6 learner needs for AI-SPs through a co-design approach; Wang et al [13] validated the feasibility of using LLMs as SPs for automated scoring; and a 2026 validation study [7] demonstrated through a 3-arm randomized controlled trial (n=58) that structurally designed AI-SPs are noninferior to human SPs in communication skill improvement.

### Implementation Barriers and User Acceptance

The successful integration of AI-SP depends not only on technological maturity but also on acceptance within the educational system. Potential barriers include the following:

1. Resistance from teachers: teachers may worry that AI-SP will replace their teaching roles or feel uneasy about its technical complexity. Coping strategies include providing low-threshold teacher training workshops, positioning the system as an “auxiliary tool” rather than a “replacement,” and inviting teachers to participate in scenario design to enhance their sense of ownership.
2. Skepticism from learners: learners may find conversations with AI “unrealistic” or worry that the system’s feedback lacks clinical relevance. Coping strategies include emphasizing its advantages (eg, unlimited repetition, immediate feedback, and judgment-free environment) during early experiences and demonstrating its effectiveness through empirical data.
3. Technical costs and infrastructure: deploying AI-SP requires certain computational support and platform development investment. For resource-constrained institutions, cloud service models or interinstitutional sharing arrangements can be considered.
4. Time competition with existing curricula: medical curricula are already highly saturated. The introduction of AI-SP should not be seen as an “additional burden” but rather as a replacement for or supplement to less efficient parts of existing teaching. It is recommended to start with elective courses or extracurricular practice, accumulate evidence, and then gradually integrate it into required courses.

## Ethical and Humanistic Considerations

The technical feasibility of AI-SP should not obscure the ethical challenges it brings [14]. This section focuses on 4 specific issues most directly relevant to AI-SP and proposes actionable design or regulatory recommendations for each.

### Revisiting the Claim of “No Risk to Real Patients”

A common claim is that AI-SP allows learners to practice “without causing risk to real patients.” This claim requires careful examination, as it may create risks of overreliance. Learners may become accustomed to interacting with AI, which in some respects (eg, the perception of nonverbal cues), is “simpler” than interacting with real patients, leading to insufficient adaptability when facing real patients. Second, there is a risk of incorrect learning. If certain responses contain inaccurate or inappropriate statements, learners may internalize them as “correct communication methods.” Finally, there are privacy and data risks. If learners’ dialogue data are improperly collected or used, sensitive information may be disclosed.

Therefore, AI-SP should be used under supervised conditions as a supplement and a preparation tool for real patient contact, not as a replacement.

### Algorithmic Bias and Clinical Authenticity

LLMs may inadvertently reflect or amplify biases present in the training data. In the medical education context, this may lead to the following problems:

1. Stereotyping: generating stereotypical descriptions or responses for patient roles from specific regions, ages, educational backgrounds, or disease states.
2. Cultural insensitivity: inability to generate responses consistent with local norms for communication scenarios involving cultural customs, religious beliefs, or value judgments. Coping strategies include explicitly incorporating doctor-patient communication norms and humanistic consensus from the Chinese context into model training and prompt design; establishing a regular review mechanism involving medical humanities experts and cross-cultural communication researchers to sample and evaluate the appropriateness of AI-SP responses; and prioritizing human review for high-risk or culturally sensitive scenarios (eg, those involving reproductive decisions or end-of-life care).

In addition, AI-SP faces a deeper issue which is clinical authenticity, that is, whether the patient roles, symptom descriptions, and emotional responses simulated by the system can truly reflect patient characteristics and needs in clinical practice. If AI-SP’s responses are overly templated or deviate from real clinical situations, learners may acquire inappropriate communication patterns or experience adaptation difficulties when facing real patients.

### Data Privacy, Informed Consent, and Legal Risks

Each interaction with AI-SP generates dialogue records between learners and patient roles. These data not only have educational assessment value but also involve complex privacy and legal issues [15]. China has not yet issued laws or regulations specifically addressing the use of AI data in medical education scenarios [16], and the standards and procedures for data anonymization remain unclear. Judicial practice has already warned that using insufficiently anonymized patient information for teaching dissemination may constitute a privacy infringement. In the AI-SP context, if a virtual patient’s identity markers are based on real cases, they may still be linked to specific individuals through information piecing, facing similar risks.

The above risks raise a series of core questions that need to be addressed: do learners know how their dialogue data are recorded, stored, and used? Will the data be used for model training? How are access permissions managed? Do learners have the right to access or delete their own data?

To address these issues, a 3-level protection mechanism is recommended. At the student level, provide clear, concise, informed-consent explanations before the first use, specifying data usage, retention period, dissemination restrictions, and data destruction procedures. At the management level, establish an “anonymized storage+tiered access+log tracking” protection mechanism with differentiated access permissions. At the content level, conduct compliance reviews of AI-SP’s teaching content, including whether virtual patient symptom descriptions are based on anonymized data, whether system responses contain inappropriate suggestions, and whether algorithmic discrimination exists, ensuring educational content compliance.

### The Necessity of Educator Oversight

Regardless of technological maturity, educator oversight is necessary for the foreseeable future. This is not only to address potential technical errors but also to ensure that system use aligns with educational goals.

Specific forms of oversight may include (1) content audits: teachers periodically sample AI-SP responses in specific scenarios to assess their accuracy, appropriateness, and consistency; (2) anomaly reporting: the system automatically flags potentially problematic interactions (eg, involving unsafe medical advice) for teacher review; (3) learner feedback channels: allow learners to mark responses that feel “unrealistic” or “potentially problematic” after interactions; and (4) teaching intervention: when systematic deviations occur in critical scenarios, teachers can quickly adjust prompts or parameters or temporarily switch to human-answered mode.

Retaining educator oversight and intervention capacity is prudent and necessary until the safety and effectiveness of AI-SP are fully validated.

## Discussion and Limitations

### Main Contributions of This Paper

Compared with the existing literature, the main contributions of this paper are reflected in the following 3 aspects.

First, it proposes an integrated technology, pedagogy, and ethics framework. Unlike studies that focus solely on technical feasibility, this paper positions the system as a “pedagogical agent” and systematically elaborates on it across 4 dimensions: system architecture, teaching theory, evaluation roadmap, and ethical considerations.

Second, it explicitly addresses the Chinese context in medical education. Based on the latest empirical data (from 506 medical education institutions and cross-cultural communication comparative studies) and national policy documents, it elucidates the special needs and conditions for developing AI-SP in the Chinese context.

Third, it proposes a testable evaluation roadmap. Unlike articles that remain at the level of conceptual advocacy, this paper proposes specific research designs and measurement tools based on the Kirkpatrick model, providing actionable starting points for subsequent empirical research.

### Educational Value of AI-SP: Beyond Experiential Learning and Deliberate Practice

The educational value of the AI-SP system proposed in this study can be further interpreted from the perspective of learner autonomy. Kolb’s experiential learning theory and Ericsson’s framework of deliberate practice explain fundamental mechanisms underlying skill acquisition. However, generative AI–supported simulation environments introduce possibilities that extend beyond the traditional “practice-feedback” cycle.

First, learners can autonomously define their learning objectives. The proposed system allows learners to select patients with different personality profiles for communication training and adjust the training difficulty according to their individual needs. This design aligns with the theoretical assumptions of self-directed learning, in which learners gain greater control over the learning process within a safe simulated environment. Such autonomy represents a core component of adult learning theory.

Second, dynamic adaptation enables personalized learning trajectories. The prompt engineering architecture allows AI-SPs to adjust their emotional responses in real time according to learners’ communication behaviors (eg, emotional relief after empathic responses from learners). This adaptive process extends beyond the “fixed task–repeated practice” paradigm traditionally emphasized in deliberate practice frameworks and provides a technological foundation for individualized learning experiences.

Third, human-AI interaction supports collaborative knowledge construction. The evaluation module implemented through the RAG+agent architecture not only provides scores but also identifies specific dialogue segments and generates targeted improvement suggestions. This enables learners to reflect on their communication assumptions and recognize potential blind spots rather than simply receiving binary judgments of “correct” or “incorrect.” Such a design aligns with the constructivist learning perspective that knowledge is actively constructed through interaction.

### Limitations of a Viewpoint Article

This paper is a viewpoint article rather than an empirical research study; therefore, the framework, system architecture, and evaluation roadmap proposed herein await subsequent empirical testing. It should be noted that our research team has conducted preliminary exploratory applications around this framework, and the initial results support its feasibility. Relevant empirical data will be presented in follow-up studies. Furthermore, because this is a conceptual framework, the description of the system architecture in this paper remains at the module level, and the discussion of user acceptance is based primarily on literature analysis and preliminary practice, lacking large-scale systematic user studies. These limitations are inherent to viewpoint articles. The goal of this paper is to propose a testable framework, not to provide a fully validated solution.

Furthermore, the current AI-SP system primarily adopts prompt engineering for patient role simulation rather than model fine-tuning. This technical choice supports dynamic persona configuration and diverse clinical scenario generation; however, it also means that role consistency remains dependent on the quality of prompt design. In complex or unexpected interaction scenarios, the system may exhibit reduced role consistency, such as generating responses containing medical knowledge beyond the predefined patient role or inconsistent emotional responses. With the accumulation of high-quality doctor-patient communication datasets, future fine-tuning approaches may further enhance the realism, stability, and personalization of AI-SP personas.

### Future Research Directions

Based on the above limitations, this paper proposes the following future research directions:

1. Technical validation studies: develop prototype systems to validate personality consistency, affective response accuracy, and medical safety in simulated consultations.
2. Teaching effectiveness evaluation: following the Kirkpatrick roadmap proposed in the *Evaluation Research Framework* section, conduct randomized controlled trials to compare differences between AI training groups and human training groups in communication skills, empathy, and self-efficacy.
3. Implementation science research: conduct implementation research in real medical schools to identify key facilitators and barriers to successful integration, and explore adaptation models under different resource conditions (Eastern vs Western and resource-rich vs resource-constrained).
4. Ethical framework refinement: based on ethical issues arising from practical use, continuously refine specific operational norms for data privacy, algorithmic bias, and educator oversight.

### Conclusions

The generative AI–SP is not a replacement for traditional medical simulation teaching but rather a conceptual evolution and extension; it reconfigures the teaching relationship among learners, educators, and simulated patients. By integrating generative AI, affective computing, and personality modeling into controllable, repeatable simulated environments, it provides new possibilities for communication training, humanistic education, and emotional competence development in medical education.

The realization of these possibilities depends on the joint satisfaction of 3 conditions: technical robustness, pedagogical adaptability, and ethical prudence. This paper proposes a “future learning” framework, whose 3 pillars are personalization, contextualization, and emotional engagement. Regarding the technical pathway, this study adopts prompt engineering as the primary approach, supplemented by RAG+agent architectures to support evaluation functions. This choice is based on the system’s educational positioning as a dynamic role-playing environment rather than standardized dialogue generation, rather than representing a technological limitation. Future improvements may incorporate model fine-tuning as sufficient high-quality interaction data become available.

This paper does not advocate replacing real patient contact or the value of human SPs with AI-SPs. On the contrary, their value lies in preparation, reflection, and supplementation: providing safe, controllable practice opportunities before contact with real patients; providing structured reflective support after contact with real patients; and providing scalable supplementary solutions in resource-limited environments.

In the intelligent era, the humanistic core of medical education will not automatically dissolve with technological intervention. How to make AI serve, rather than replace, the humanistic dimensions of the doctor-patient relationship is a discussion that this paper hopes to initiate, not a definitive answer that it provides. Ultimately, whether technology can truly serve humanistic values depends on how educators design, implement, and critically evaluate its use, and this remains a fundamental and enduring question in medical education.

## Supplementary material

10.2196/91050 Multimedia Appendix 1 Technical architecture and workflow of the AI-driven digital standardized patient prototype system.

## Abbreviations

AI-SP: AI-driven digital standardized patient
LLM: large language model
OSCE: objective structured clinical examination
RAG: retrieval-augmented generation
SEGUE: Set the Stage, Elicit Information, Give Information, Understand the Patient’s Perspective, and End the Encounter
SP: standardized patient
SPIKES: setting up the interview, assessing the patient's perception, obtaining the patient's invitation, giving knowledge and information, addressing empathy and emotions, strategy and summary
